# Rifabutin central nervous system concentrations in a rabbit model of tuberculous meningitis

**DOI:** 10.1128/aac.00783-24

**Published:** 2024-07-19

**Authors:** Sean Wasserman, Rosleine Antilus-Sainte, Noha Abdelgawad, Narineh M. Odjourian, Melissa Cristaldo, Maureen Dougher, Firat Kaya, Matthew Zimmerman, Paolo Denti, Martin Gengenbacher

**Affiliations:** 1 Institute for Infection and Immunity, St. George’s, University of London, London, United Kingdom; 2 Center for Infectious Diseases Research in Africa, Institute of Infectious Disease and Molecular Medicine, University of Cape Town, Cape Town, South Africa; 3 Center for Discovery and Innovation, Hackensack Meridian Health, Nutley, New Jersey, USA; 4 Division of Clinical Pharmacology, Department of Medicine, University of Cape Town, Cape Town, South Africa; 5 Hackensack Meridian School of Medicine, Nutley, New Jersey, USA; Bill & Melinda Gates Medical Research Institute, Cambridge, Massachusetts, USA

**Keywords:** *Mycobacterium tuberculosis*, tuberculous meningitis, rifamycins, site-of-disease pharmacokinetics, preclinical models

## Abstract

Tuberculous meningitis (TBM) has a high mortality, possibly due to suboptimal therapy. Drug exposure data of antituberculosis agents in the central nervous system (CNS) are required to develop more effective regimens. Rifabutin is a rifamycin equivalently potent to rifampin in human pulmonary tuberculosis. Here, we show that human-equivalent doses of rifabutin achieved potentially therapeutic exposure in relevant CNS tissues in a rabbit model of TBM, supporting further evaluation in clinical trials.

## INTRODUCTION

Tuberculous meningitis (TBM) affects an estimated 150,000 people annually and is the most lethal form of tuberculosis (1). Mortality is up to 40% and many survivors experience permanent disability, despite treatment with antituberculosis therapy (2). One reason for this poor treatment response is that standard chemotherapy for TBM (rifampin, isoniazid, pyrazinamide, and ethambutol) is the same as for pulmonary TB, where, unlike TBM, disease is located outside the central nervous system (CNS), mortality is low, and the treatment goal is to prevent relapse. TBM treatment is therefore not optimized for CNS infection and may not achieve therapeutic concentrations at the site of disease. In TBM patients, total (protein-bound plus unbound) cerebrospinal fluid (CSF) concentrations of the key drug, rifampin, are 10- to 20-fold lower than in plasma (3, 4), and brain exposures are variable and spatially heterogenous in animal models (5). Higher doses of rifampin provide CSF exposures (3, 6) and may improve clinical outcomes (7), which is currently being evaluated in definitive trials. However, new approaches for optimizing TBM regimens should also be explored, combining agents with potent antituberculosis activity and enhanced CNS penetration.

Rifabutin, a rifamycin agent, has several favorable characteristics supporting potential use as an alternative to rifampin for TBM therapy. It has a much lower minimum inhibitory concentration (MIC) against *Mycobacterium tuberculosis*, distributes widely *in vivo*, and concentrates within host cells (8, 9). Rifabutin is associated with more rapid mycobacterial clearance in both pulmonary tuberculosis preclinical models (10) and in patients (11), and it had similar efficacy to rifampin in clinical trials for pulmonary tuberculosis (12). The potential use of rifabutin for treatment of meningitis is supported by its efficacy in a rabbit model of pneumococcal meningitis (13). More importantly, rifabutin has a much weaker effect on cytochrome P450 metabolism than rifampin and can be co-administered with bedaquiline, offering an opportunity for combining them in novel TBM regimens. Data confirming rifabutin exposure at site of disease for TBM are required prior to evaluation in clinical trials. We performed a preclinical pharmacokinetic (PK) study to describe rifabutin CNS concentrations in an infected rabbit model of TBM.

Our New Zealand white rabbit TBM model is optimized to recapitulate human TBM disease with features including variable duration of symptom onset, typical clinical manifestations, representative pathology, and compatible radiological features (14). Animal studies were approved by the Hackensack Meridian Health Institutional Animal Care and Use Committee. Rabbits were infected with 10^4^ CFUs Mtb HN878 via the cisterna magna (14, 15) and treated with rifabutin once daily at 15 mg/kg (equivalent to human doses of 300 mg) for 3 days by oral gavage after reaching a predefined neurological score. Rifabutin was formulated in 0.5% carboxymethyl cellulose/0.5% Tween 80/sterile water (16). Blood was collected from the central ear artery pre-dose and at 0.5, 1, 2, 3, 5, 6, 7, 10, and 24 h post-drug administration on day 1 and until the time of necropsy on day 3. Rabbits were necropsied, with a terminal CSF sample collected, at either 3, 6, 10, or 24 h after the third dose. The brain, meninges, cervical and lumbar spinal cord, and lung were collected for total rifabutin (protein-bound plus unbound) quantification by liquid chromatography-mass spectrometry as previously described (17). Noncompartmental analysis was performed to describe secondary PK parameters using PK Solver, a Microsoft Excel add-in (18), and figures were produced in R.

**Fig 1:**
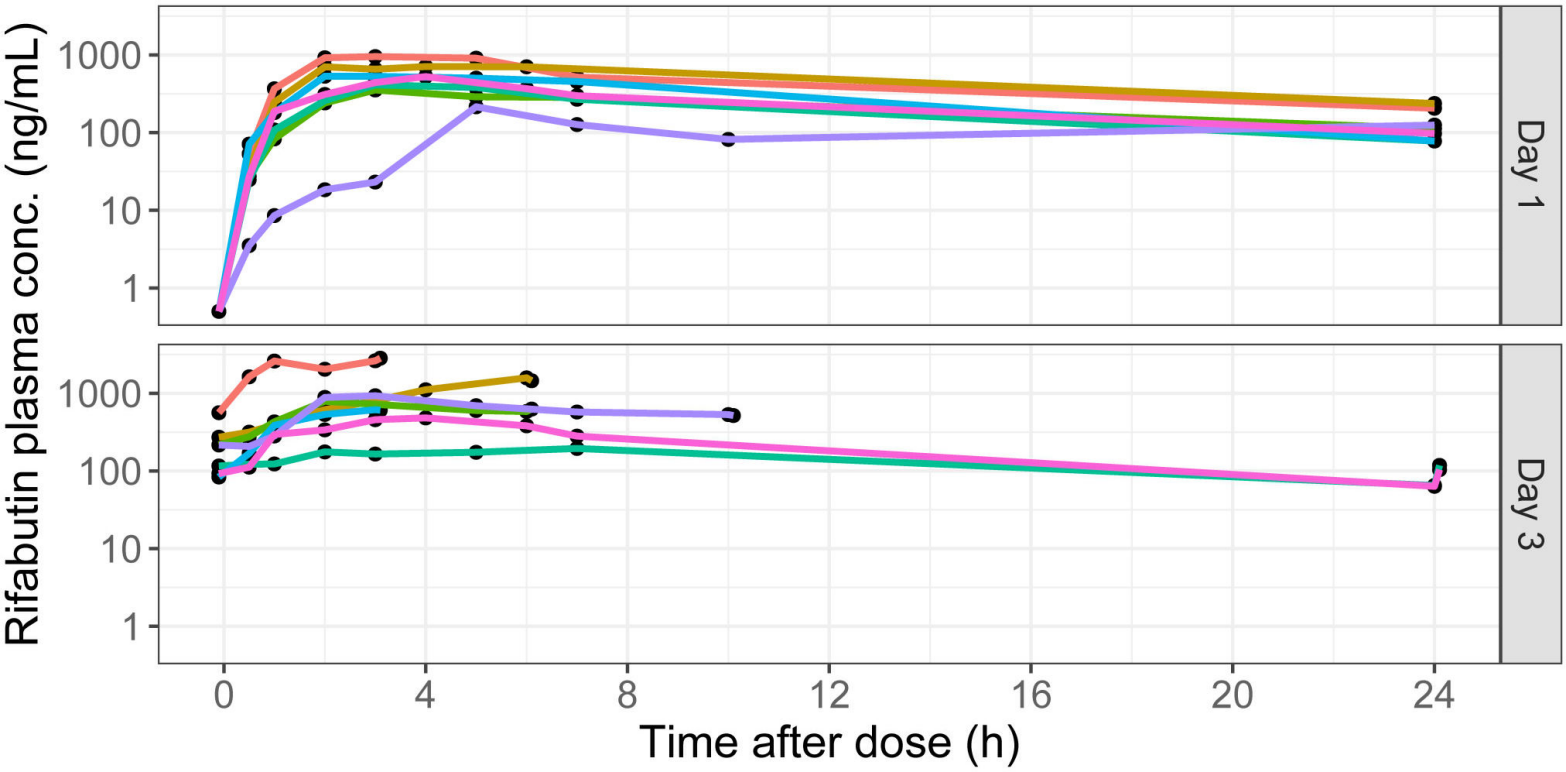
Plasma concentrations of rifabutin in TBM rabbits at 1 and 3 days of dosing. Plasma was isolated from 0.5 mL of whole blood collected from the ear artery at designated time points. Rifabutin was quantified by liquid chromatography-mass spectrometry. At all terminal time points blood from each animal was sampled two times, at 3, 6, 10, or 24 h post dosing on day 3 and ~10 min thereafter immediately prior tp tissue collection. Color-coded graphs of individual animals are shown.

**Fig 2:**
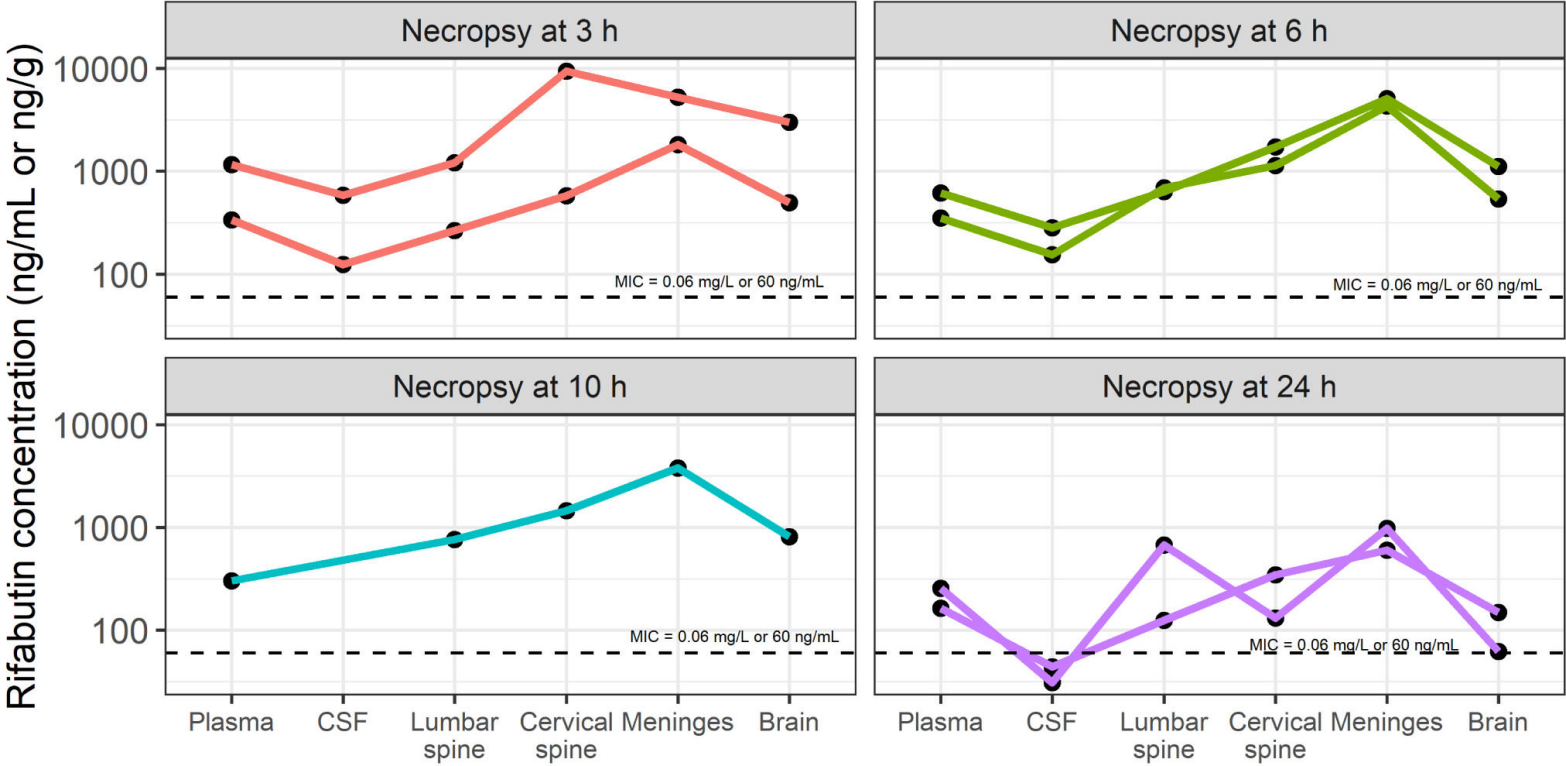
Rifabutin in most target tissues remains above the critical concentration over the dosing interval. Rabbits having TBM with a neurological score of 3 received three daily doses of rifabutin at 15 mg/kg (human-equivalent dose) by oral gavage. After the third and final dose, animals were euthanized at 3, 6, 10, and 24 h for drug quantification in tissues and body fluids. Connected data points represent individual rabbits.

Seven rabbits were included with median weight of 3.27 kg, providing 58 rifabutin plasma concentrations from full sampling up to 24 h post-dose on days 1 and 7 paired plasma, CSF and tissue concentrations from terminal sampling on day 3. Summary statistics are provided in Table 1. Plasma observations sampled on days 1 and 3 are depicted in Fig. 1. For day 1 plasma profiles, the median (first to third quartiles) concentration was 281 (110–532) ng/mL, plasma rifabutin AUC_0–24_ was 5,766 (5,017–9,320) h·ng/mL, and AUC_0–infinity_ was 7,227 (6,540–11,148) in line with published plasma AUC from TB patients dosed at the standard 300 mg daily (19–21). After one CSF sample was excluded because of blood contamination, the median (first to third quartiles) concentration observed in CSF was 140 (64.2–251) ng/mL. When comparing these values with the terminal plasma sample, the CSF/plasma ratio was 0.227 (0.205–0.285). Median (first to third quartiles) brain rifabutin concentration was 536 (321–961) ng/mL, with a brain/plasma ratio of 0.856 (0.790–1.16). Concentrations across CNS compartments, plasma, and lung are shown in Fig. 2, demonstrating higher concentrations in the meninges and spinal cord relative to plasma, CSF, and other CNS tissues. Rifabutin concentrations exceeded the *in vitro* MIC for *M. tuberculosis* of 0.06 mg/L (22) in all CNS compartments and throughout the dosing interval, except for the two CSF concentrations at 24 h necropsy time.

**TABLE 1:** Pharmacokinetic parameters and tissue drug concentrations of rifabutin in TBM rabbits

Parameter	Value median (first to third quartiles)
Plasma AUC_0-24h_ on day 1, h·ng/mL	5,766 (5,017–9,320)
Plasma AUC_0-inf_ on day 1, h·ng/mL	7,227 (6,540–11,148)^*a*^
Plasma C_max_ on day 1, ng/mL	526 (383–621)
Plasma C_24h_ day 1, ng/mL	113 (87.7–166)
Terminal plasma concentration on day 3, ng/mL	607 (318–1,038)
CSF concentration, ng/mL	140 (64.2–251)
Brain concentration, ng/g	536 (321–961)
Lumbar spine concentration, ng/g	673 (452–728)
Cervical spine concentration, ng/g	1,140 (463–1,590)
Meningeal concentration, ng/g	3,770 (1,395–4,700)
Lung concentration, ng/g	8,410 (4,405–19,750)

^*a*^ For one rabbit, the elimination rate constant could not be reliably estimated, so the median of the other six rabbits (0.0709 h^−1^) was used to calculate AUC_0-inf_ of that rabbit.

These data show that, at human-equivalent doses, rifabutin achieves potentially therapeutic exposures in the CNS of rabbits with TBM. The relative penetration of rifabutin from plasma into CSF and brain tissue exceeds that of rifampin from preclinical TBM studies (5). Our observations are corroborated by other evidence. In a healthy non-human primate study, rifabutin achieved relatively high CSF concentrations with a total CSF/plasma ratio ranging between 0.29 and 0.42; when free drug was measured, this increased to 2.4–3.4 (23). Similar observations were made in a rifabutin dose ranging study among people with advanced HIV, where a daily dose of 450 mg led to mean serum and CSF concentrations of 92.5 ng/mL (range 65.5–135.6) and 46.9 ng/mL (26.5–69.9), respectively, with a penetration ratio of 0.50 (range 0.36–0.70) (24).

A fundamental limitation of preclinical studies is imperfect translation to patients. However, this approach is essential for drug evaluation in TBM because concentrations in CSF, the only CNS compartment accessible from patients, correlate poorly with brain exposure where most pathology occurs. Our rabbit TBM model is optimized to replicate human TBM disease, providing information that can support drug selection for clinical trials. We did not evaluate the efficacy of rifabutin because of rapid disease progression after symptom onset in rabbits, plus lack of predictive biomarkers for treatment response in TBM. Furthermore, we did not include a control group of rabbits with rifampin dosing, precluding direct comparison in this model.

In summary, human-equivalent doses of rifabutin achieved relatively high concentrations at the site of disease in a preclinical model of TBM. Given the potent antituberculosis activity of rifabutin and equivalent clinical efficacy to rifampin for pulmonary tuberculosis, these findings strongly support the evaluation of rifabutin in clinical trials for TBM.
